# Do Diacritical Marks Play a Role at the Early Stages of Word Recognition in Arabic?

**DOI:** 10.3389/fpsyg.2016.01255

**Published:** 2016-08-22

**Authors:** Manuel Perea, Reem Abu Mallouh, Ahmed Mohammed, Batoul Khalifa, Manuel Carreiras

**Affiliations:** ^1^Department of Methodology, Universitat de ValènciaValencia, Spain; ^2^Basque Center on Cognition, Brain, and LanguageDonostia, Spain; ^3^Psychological Sciences Department, Qatar UniversityDoha, Qatar; ^4^Ikerbasque Basque Foundation for ScienceBilbao, Spain

**Keywords:** lexical access, masked priming, visual-letter similarity, lexical decision

## Abstract

A crucial question in the domain of visual word recognition is whether letter similarity plays a role in the early stages of visual word processing. Here we focused on Arabic because in this language there are various groups of letters that share the same basic shape and only differ in the number/location of diacritical points. We conducted a masked priming lexical decision experiment in which a target word was preceded by: (i) an identity prime; (ii) a prime in which the critical letter was replaced by a letter with the same shape that differed in the number of diacritics (e.g., 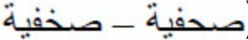); or (iii) a prime in which the critical letter was replaced by a letter with different shape (e.g., 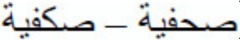). Results showed a sizable advantage of the identity condition over the two substituted-letter priming conditions (i.e., diacritical information is rapidly processed). Thus, diacritical marks play an essential role in the “feature letter” level of models of visual word recognition in Arabic.

## Introduction

As reviewed by Grainger et al. (2016), models of visual word recognition in the Roman alphabet assume that the visual form of the word's component letters is quickly mapped onto abstract units irrespective of font, case, position, or size. These abstract letter units are the driving force behind the process of visual word recognition. This analysis is consistent with the available empirical evidence. For instance, in a masked priming experiment, Jacobs et al. (1995) found that the lowercase prime judge and the uppercase prime JUDGE were equally effective at activating the target word JUDGE (i.e., there was no advantage of the physically identical condition over the nominally identical condition). In an electrophysiological experiment, Vergara-Martínez et al. (2015) replicated this behavioral phenomenon and found a difference between judge-JUDGE and JUDGE- JUDGE in an early (visual) ERP component (N/P150)—this difference completely disappeared in orthographical-lexical components (N250 and N400). Similarly, Bowers et al. (1998) found an equivalent magnitude of masked identity priming, relative to an unrelated condition, for words that look visually similar across case (e.g., kiss-KISS) and for cross-case words that look visually dissimilar across case (e.g., gale-GALE). Finally, recent research has shown that visual similarity plays some role for briefly presented primes containing letter-like digits or symbols (e.g., M4TERI4L-MATERIAL faster than M6TERI6L-MATERIAL; see Perea et al., 2008), but these effects do not seem to occur for substituted-letter primes (e.g., the visually-similar prime HRHNDON does not facilitate the processing of the target abandon more than the control prime DWDNDON; see Kinoshita et al., 2013).

In the present study, we examined whether visual-letter similarity plays a role at the early stages of word processing in another commonly-used script: Arabic. Unlike the Roman script, Arabic does not have a lowercase/uppercase distinction and it is read from right to left. In addition, some letters are connected to the following letter, but others are not—Arabic follows strict rules in this respect. Furthermore, the visual shape of the letters may look visually different depending on whether a letter is presented in isolation or in the initial, middle or final position within the letter chunk (e.g., the Arabic letter ayn looks quite different in an isolated position and in a middle position: 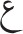 and, 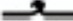 respectively). To explore the role of visual similarity in Arabic, Perea et al. (2013) conducted a series of masked priming lexical decision experiments in which primes and targets had the same (visual) ligation pattern (e.g., 
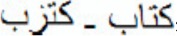
, ktzb-ktAb [book] with the Buckwalter transliteration) or not (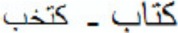, ktxb-ktAb). Results showed remarkably similar word recognition times for these two substituted-letter conditions—there was only a similar advantage of these two conditions over an unrelated priming condition (e.g., 
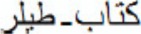
 Tylr- ktAb). Perea et al. (2013) concluded that in Arabic, as occurs with the Roman script, there is access to “abstract letter representations in the early stages of visual word recognition” (p. 572). Similarly, in a masked prime same-different experiment with individuals who mastered both the Arabic and Roman scripts, Carreiras et al. (2013) found that the electrophysiological responses for Arabic letters (middle vs. isolated form) were similar to those for Roman letters (lowercase vs. uppercase); furthermore, the visual similarity effect disappeared in the P300 component in the two scripts. In addition, Yakup et al. (2015) failed to find a relationship between the magnitude of masked form priming and an estimated measure of visual similarity in Uyghur (i.e., a language which is written with Arabic script). Thus, despite the obvious differences between the visual processing of words in the Roman and Arabic scripts, the underlying processes seem be, to some degree, analogous (see Okano et al., 2013; for a similar observation regarding Japanese Katakana).

While the above-cited findings are undoubtedly important, one could argue that none of these experiments in Arabic directly manipulated visual-letter similarity. In the present research, we focused on a highly relevant feature of Arabic script related to visual-letter similarity. Most of the 28 Arabic letters share the basic shape with at least one other letter, so that they only differ in the number (or location) of diacritical points: (1) 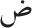 and 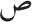 [the IPA codes are /d^ʕ^/ and /s^ʕ^/, respectively]; (2) 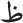 and 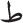 [/z^ʕ^/ and /t^ʕ^/]; (3) 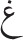 and 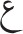 [/ɤ/ and /ʕ/]; (4) 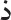 and 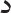 [/ð/ and /d/]; (5) 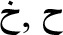 and 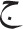. [/x/, /ħ/, and /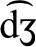/]; (6) 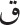 and 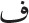 [/q/ and /f/]; (7) 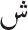 and 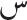 [/ʃ/ and /s/]; (8) 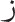 and 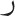 [/z/ and /r/]; and (9) 
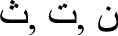
, and 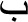 [/θ/, t /t/, /n/, and /b/]. Indeed, there was a time in the evolution of Arabic script in which the letters did not have diacritical points. That is, readers would need to deduce from context—and this was not always possible—whether a letter like 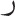 would correspond to /z/ or /r/. Around the 7th century, diacritical dots were added to the Arabic script to avoid this ambiguity, thus creating the letters for Classical Arabic. As a result, it is possible to create pairs of stimuli in Arabic that look visually very similar—i.e., the basic shape would be exactly the same. This allowed us to directly manipulate visual-letter similarity. As in prior research, we employed a masked priming lexical decision task. For each target word (e.g., 
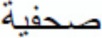
 SHfyp [journalist]), we created two substituted-letter primes: (a) a prime in which one of the letters from the target was replaced by another letter with the same basic shape (i.e., the only difference was the number of the diacritical points; 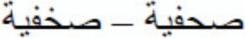 Sxfyp- SHfyp; *same-shape substituted-letter prime*); and (b) a prime in which the critical letter was replaced by another letter with a different basic shape (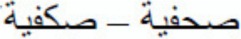 Skfyp- SHfyp; *different-shape substituted-letter prime*). As in previous research (e.g., Perea et al., 2013), we also included an identity priming condition for comparison purposes. Substituted-letter primes were always nonwords and involved the replacement of a root letter—note that Perea et al. (2014) reported a small but significant masked form priming effect in Arabic.

The predictions are the following: If we assume that early in word processing, there is some degree of uncertainty for those letter identities that share the basic shape, same-shape substituted-letter primes would enjoy some advantage over different-shape substituted-letter primes (e.g., 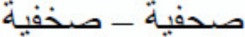 – faster than 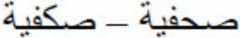). Alternatively, if diacritical marks are assembled at the very early stages of word processing, each of the letters that compose the stimulus would only activate its corresponding best-letter match—in this scenario, we would only expect an advantage of the identity condition over the two substituted-letter priming conditions.

## Materials and methods

### Participants

Twenty-seven university students from Qatar University participated voluntarily in the experiment. All of them were native speakers of Arabic with normal or corrected-to-normal vision. The Ethics Committee of Qatar University approved this experiment. All participants gave written informed consent in accordance with the Declaration of Helsinki.

### Materials

We selected 264 Arabic words of five letters, all of them with productive roots, from the Aralex database of Modern Standard Arabic (Boudelaa and Marslen-Wilson, 2010). The mean frequency per million in the Aralex database was 11.7 (range: 0.01–255.5). All the stimuli had a critical pairs of letters that shared the basic shape: 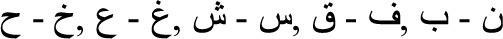, and 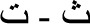 Each target word was preceded by a prime stimulus that was: (a) identical to the target (e.g., 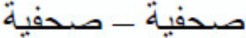 SHfyp- SHfyp; identity condition); (b) identical to the target except for the substitution of a root letter with another letter that kept the basic shape (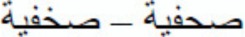 Sxfyp- SHfyp; same-shape substituted-letter condition); or (c) the same as the target except for the substitution of the critical letter with another letter with a different basic shape while keeping the ligation pattern (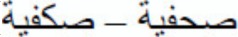 Skfyp- SHfyp; different-shape substituted-letter condition). We prepared three lists to rotate the target words across the three priming conditions in a Latin square design—there were 88 items/condition. We also created a set of 264 nonwords of five letters in Arabic to be used as foils in the lexical decision task. These nonwords have been created by replacing one or two letters from an Arabic word (e.g., the nonword 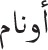 >wnAm was created by changing one letter from the word 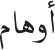 >wham [*illusions*]). The prime-target manipulation was the same as that for word targets. Nine participants were randomly assigned to each list. The set of stimuli is available at: http://www.uv.es/mperea/DiacriticsArabic.pdf

### Procedure

The experimental session took place individually in a silent lab. To present the stimuli and collect the participant responses, we used a Windows-OS computer equipped with DMDX (Forster and Forster, 2003). All the stimuli (mask, prime, target) were displayed in the same spatial location at the center of the screen. The setup of a given trial was the following: (a) a mask composed of #'s was presented for 500 ms; (b) the prime was presented for 50 ms; and (c) the target was presented until the participant responded or 2 s had passed. To prevent visual continuity, the font size of the prime (DejaVu Sans Mono 14-pt; i.e., a fixed-width font) was smaller than that of the target (DejaVu Sans Mono 28-pt; see Perea et al., 2013, 2014). Participants were instructed to press the “yes” button if the letter string was an Arabic word and to press the “no” button otherwise. They were asked to make this decision as rapidly and as accurately as possible. Participants were not informed about the presence of briefly presented stimuli, and they did not report having seen them when asked after the experiment. Sixteen practice trials preceded the 528 experimental trials—the order of experimental trials was fully randomized for each participant. The whole session lasted for around 21–25 min.

## Results

Response times (RTs) shorter than 250 ms were removed from the correct RT analyses. The mean correct RTs and accuracy for word and nonword targets in each experimental condition (identity condition, same-shape substituted letter prime; different-shape substituted letter prime) are displayed in Table 1.

**Table 1:** **Mean response times (in ms) and accuracy (in parentheses) for words and nonwords in the three prime-target conditions of the experiment**.

	**Identity**	**Same shape substituted-letter**	**Different shape substituted-letter**
Words	758 (0.898)	777 (0.890)	775 (0.895)
Nonwords	869 (0.822)	883 (0.803)	868 (0.825)

The correct RT data were analyzed using linear mixed-effects models in R (R Core Team, 2016). For the word trials, there were 6375 data points in the RT analyses—for the nonword trials, the data points were 5819. As RT distributions show positive asymmetry, raw RTs were inverse-transformed (−1000/RT) to maintain the Gaussian assumption of liner mixed-effects models. The coding of the fixed factor “Prime-target relationship” tested the two contrasts of interest: (a) identity prime vs. same-shape substituted-letter prime; and (b) same-shape substituted-letter prime vs. different-shape substituted-letter prime. We employed the package *lme4* (Bates et al., 2015) to obtain *t*-values, and the package *lmerTest* (Kuznetsova et al., 2016) to obtain the corresponding *p*-values. With respect to the random effects, we employed the maximal random effects structure model. That is, the resulting model for the latency analyses of word trials was the following: LME_WordRTs = lmer(inv_RT ~ type_of_prime + (type_of_prime +1|item) + (type_of_prime +1|subject), data = WordRTs)—the model for the nonword trials was parallel except that we had NonwordRTs instead of WordsRTs. The accuracy data were modeled using the glmer function (family = binomial) and the data were coded as binary values (0 vs. 1). From the glmer summary command, we obtained z-values and their corresponding *p*-values.

### Word data

The lexical decision data showed a 19-ms advantage of the identity condition over the same-shape substituted-letter conditions, coefficient = −0.0368, *SE* = 0.0103, *t* = −3.59, *p* < 001, while there were no signs of a difference between the same-shape and different-shape substituted-letter conditions, coefficient = −0.0043, *SE* = 0.0126, *t* = 0.34, *p* > 0.73. Finally, a post hoc analysis showed that the 17 ms advantage of the identity condition over the different-shape substituted-letter condition was statistically significant, coefficient = −0.00326, *SE* = 0.0101, *t* = 3.22, *p* = 0.002.

The analyses of the accuracy data failed to find any significant effects, both |*z*s| < 1.

### Nonword data

The analyses of the RT data for nonwords did not show any trends of an effect, both |*t*s| < 1. The analyses of the accuracy data only showed a small (1.8%) advantage of the identity condition over the same-shape substituted-letter condition that approached significance, coefficient = −0.16065, *SE* = 0.08215, *z* = 1.95, *p* = 0.051, whereas there were no signs of a difference between same-shape and different-shape substituted-letter conditions, |*z*| < 1.

## Discussion

The current masked priming lexical decision experiment showed that substituted-letter primes in which the replaced letter kept the same basic shape as the original letter (e.g., 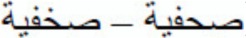) were not more effective at activating the base words than substituted-letter primes in which the replaced letter was visually different (e.g., 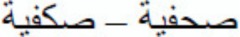) (i.e., a −2 ms difference). The null effect for the two substituted-letter primes was not due to lack of processing of the primes, as we found a significant 19-ms advantage of the identity condition over the same-shape substituted-letter priming condition.

These findings support the view that there is fast access to abstract representations in Arabic (see Carreiras et al., 2012, 2013; Perea et al., 2013): each letter (e.g., 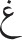) activates quickly its corresponding letter representation so that the visually similar letter 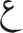 is no more effective in activating its abstract representation than a visually dissimilar letter (e.g., 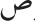). Another important implication of the present experiment is that diacritical information is highly relevant in the “letter feature” level of Arabic, as it is key to distinguishing among many letters that otherwise share the same basic shape. Keep in mind that, as indicated in the Introduction, the majority of Arabic letters share their basic shape with at least one other letter. Failing to correctly identify the right letter would necessarily produce a reading cost. Consistent with this view, in a recent study with pairs of isolated letters in Arabic, Wiley et al. (2016) found that the diacritic marks were the “the most important feature (…) for both discrimination time and accuracy.”

Current neural and computational models of visual word recognition have focused on the Roman alphabet, and more specifically, on the orthographic and lexical levels. A limitation of these models is the lack of a detailed specification of the “letter feature” level and how this level is mapped onto the “abstract” letter level—for instance, current computational models of visual word recognition still use the unrealistic uppercase font devised by Rumelhart and Siple (1974) (see Blais et al., 2009; Davis, 2010; Rosa et al., 2016, for discussion). The special characteristics of the Arabic alphabet raises a number of questions (e.g., position-dependent allography, diacritical marks, and its cursive nature) that may help implement a more comprehensive model of visual word recognition in alphabetic languages.

In sum, diacritical marks in Arabic are the only visual element that distinguishes a large percentage of Arabic letters. The present experiment demonstrated that this information is rapidly processed by the cognitive system, even when the prime stimulus is presented very briefly (50 ms) and is forwardly and backwardly masked. Therefore, at the early stages of word processing in Arabic, the cognitive system of adult readers is able to distinguish between identity priming condition (e.g., 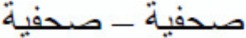) and a same-shape substituted-letter condition that only varies in the number of diacritics (e.g., 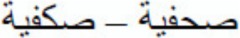). Further research should examine whether developing readers can tell apart these seemingly visually-similar words early in processing and whether reading skill (e.g., good vs. bad readers) modulates this process.

## Author contributions

MP, MC, RA, BK, and AM designed the experiment. RA prepared the materials. AM conducted the experiment. MP and MC analyzed the data. MP and MC wrote the initial draft.

## Funding

The research reported in this article has been supported by Grant NPRP No. 6-378-5–035 from Qatar Foundation.

### Conflict of interest statement

The authors declare that the research was conducted in the absence of any commercial or financial relationships that could be construed as a potential conflict of interest.
